# Clarithromycin Modulates Neutrophilic Inflammation Induced by *Prevotella intermedia* in Human Airway Epithelial Cells

**DOI:** 10.3390/antibiotics13090909

**Published:** 2024-09-23

**Authors:** Naoki Iwanaga, Ayaka Ota, Hiroki Ashizawa, Yuya Ito, Tatsuro Hirayama, Masataka Yoshida, Kazuaki Takeda, Shotaro Ide, Masato Tashiro, Naoki Hosogaya, Noriho Sakamoto, Takahiro Takazono, Kosuke Kosai, Mariko Naito, Yoshimasa Tanaka, Kazuhiro Yatera, Koichi Izumikawa, Katsunori Yanagihara, Hiroshi Mukae

**Affiliations:** 1 Department of Respiratory Medicine, Nagasaki University Hospital, Nagasaki 852-8501, Japan; hiroashi@nagasaki-u.ac.jp (H.A.); yuya.ito@nih.gov (Y.I.); m-yoshida@nagasaki-u.ac.jp (M.Y.); k-takeda@nagasaki-u.ac.jp (K.T.); nhosogaya@nagasaki-u.ac.jp (N.H.); nsakamot@nagasaki-u.ac.jp (N.S.); takahiro-takazono@nagasaki-u.ac.jp (T.T.); hmukae@nagasaki-u.ac.jp (H.M.); 2 Department of Pharmacotherapeutics, Nagasaki University Graduate School of Biomedical Sciences, Nagasaki 852-8501, Japan; bb30219004@ms.nagasaki-u.ac.jp (A.O.); tatsuro_h_20@nagasaki-u.ac.jp (T.H.); 3 Department of Respiratory Medicine, Nagasaki University Graduate School of Biomedical Sciences, Nagasaki 852-8501, Japan; 4 Laboratory of Clinical Immunology and Microbiology, National Institute of Allergy and Infectious Diseases, National Institutes of Health (NIH), Bethesda, MD 20892, USA; 5 Department of Infectious Diseases Experts Training Center, Nagasaki University Hospital, Nagasaki 852-8501, Japan; str-ide@nagasaki-u.ac.jp; 6 Department of Infectious Diseases, Nagasaki University Graduate School of Biomedical Sciences, Nagasaki 852-8501, Japan; mtashiro@nagasaki-u.ac.jp (M.T.); koizumik@nagasaki-u.ac.jp (K.I.); 7 Clinical Research Center, Nagasaki University Hospital, Nagasaki 852-8501, Japan; 8 Department of Laboratory Medicine, Nagasaki University Hospital, Nagasaki 852-8501, Japan; k-kosai@nagasaki-u.ac.jp (K.K.); k-yanagi@nagasaki-u.ac.jp (K.Y.); 9 Department of Microbiology and Oral Infection, Nagasaki University Graduate School of Biomedical Sciences, Nagasaki 852-8501, Japan; mnaito@nagasaki-u.ac.jp; 10 Center for Medical Innovation, Nagasaki University, Nagasaki 852-8588, Japan; ystanaka@nagasaki-u.ac.jp; 11 Department of Respiratory Medicine, University of Occupational and Environmental Health, Kitakyushu 807-8555, Japan; yatera@med.uoeh-u.ac.jp

**Keywords:** *Prevotella intermedia*, clarithromycin, human airway epithelial cells

## Abstract

**Objectives:** In the present study, we aimed to clarify the mechanisms by which periodontal pathogens, particularly *Prevotella intermedia*, induce severe neutrophilic inflammation. In addition, we aimed to test the efficacy of macrolides, which has not been resolved in the neutrophilic inflammation induced by *P. intermedia*. **Methods:** NCl-H292 human airway epithelial cells were pre-incubated with clarithromycin for 2 h before incubation with *P. intermedia* supernatants. Then, C-X-C motif chemokine ligand 8 (*CXCL8*) transcription and interleukin (IL)-8 production were measured. To elucidate the signaling pathway, mitogen-activated protein kinase inhibitors were added to the cell culture, and the cells were subjected to Western blotting. **Results:** *P. intermedia* supernatants promoted *CXCL8* transcription and IL-8 production, and the reactions were significantly suppressed by clarithromycin pretreatment. Only trametinib, the selective mitogen-activated extracellular signal-regulated kinase inhibitor, downregulated *CXCL8* transcription and IL-8 production. Furthermore, Western blotting revealed that stimulation with *P. intermedia* supernatants specifically induces extracellular signal-regulated kinases (ERK) 1/2 phosphorylation, which is suppressed by clarithromycin pretreatment. Notably, the interference analysis revealed that ERK3 might be dispensable for IL-8 production under the stimulation of *P. intermedia* supernatants. **Conclusions:** Our results provide new insight into the mechanism underlying *P. intermedia*-induced production of IL-8 from human airway epithelial cells. Furthermore, macrolides might have therapeutic potential in regulating periodontal pathogen-induced neutrophilic inflammation in the lungs.

## 1. Introduction

Periodontal pathogens, such as *Prevotella intermedia* (*P. intermedia*), cause periodontitis through evasion mechanisms of the host immune response and by establishing themselves in the periodontal region [1]. Notably, *P. intermedia* suppresses polymorphonuclear neutrophil (PMN) activity through human dental follicle stem cells, reducing PMN-induced tissue and bone deterioration and allowing the survival of oral pathogens [2]. Endodontic pathogens, such as *Fusobacterium nucleatum*, *Parvimonas micra*, and *P. intermedia*, have been reported to efficiently neutralize and kill infiltrating neutrophils in a mouse subcutaneous infection model [3]. Furthermore, periodontal pathogens secrete extracellular deoxyribonucleases that degrade DNA/chromatin filaments known as neutrophil extracellular traps, allowing them to evade host immune defenses [4]. Notably, *P. intermedia* has the highest DNA degradation activity [4,5]. Furthermore, *Prevotella* spp. has been suggested to stimulate epithelial cells to produce interleukin (IL)-8, IL-6, and chemokine (C-C motif) ligand 20 (CCL20) and promote mucosal T helper (Th) 17 immune responses and neutrophil recruitment in cases of chronic lung infection, causing the systemic spread of inflammatory mediators, bacteria, and bacterial products, which may influence systemic disease outcomes, such as rheumatoid arthritis and metabolic disorders [6]. In addition, the chemotaxis of excessive neutrophils to the infection site plays a crucial role in biofilm formation [7] and contributes significantly to the establishment of persistent *Pseudomonas aeruginosa* (*P. aeruginosa*) infection in patients with cystic fibrosis [8,9] and to the emergence of drug resistance in cases of *P. aeruginosa* [10]. Generally, in cases of bronchiectasis, the abundant neutrophilic infiltration into the lungs is critical to the pathophysiology, and the relative contribution of proteases derived from neutrophils, cytokines, and inflammatory mediators to the spread of inflammation has been reported [11]. Notably, it has been reported in recent clinical studies that *P. intermedia* might cause pneumonia as a mixed infection with oral streptococcus [12]. In preclinical studies, the introduction of *P. intermedia* culture supernatants with pneumococcus was detrimental to pneumonia, presenting severe neutrophilic inflammation in the lungs [13].

Macrolide antimicrobials have been reported to exert potent immunomodulatory effects in cases of various inflammatory lung diseases, particularly those in which excessive neutrophils are present, such as diffuse panbronchiolitis [14]. Their immunomodulatory effects and mechanisms of action vary depending on the clinical setting. Their efficacy in cases of acute severe pneumonia, in which high inflammation and immunosuppression occur simultaneously, has been reported recently [15]. Nagaoka et al. reported that macrolides inhibited the phosphorylation of extracellular signal-regulated kinase 1/2 (ERK1/2) induced by the culture supernatants of *F. nucleatum* and reduced MUC5AC production [16]. However, the efficacy and mechanisms of macrolides on neutrophilic inflammation induced by *P. intermedia* have yet to be clarified.

In the present study, we aimed to clarify the mechanisms through which periodontal pathogens, particularly *P. intermedia*, induce severe neutrophilic inflammation. In addition, we aimed to promptly test the efficacy of macrolide-based drugs. This study might be crucial in the context of refractory pulmonary infection cases in which excessive neutrophils were present due to *Prevotella* spp. and shed light on the potential roles of macrolides.

## 2. Results

### 2.1. Clarithromycin Suppressed C-X-C Motif Chemokine Ligand 8 Messenger RNA Expression and IL-8 Production Induced by Prevotella intermedia Supernatants

**Figure 1:**
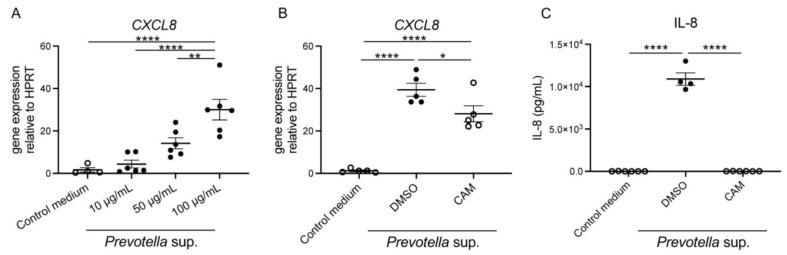
Enhancement of *CXCL8* mRNA expression and IL-8 production by *P. intermedia* supernatants and inhibition of the reactions by clarithromycin pretreatment. (**A**) *CXCL8* mRNA expression in H292 cells was assessed using RT-PCR under co-incubation with *P. intermedia* supernatants for 4 h. *P. intermedia* supernatants enhanced *CXCL8* mRNA expression in a protein concentration-dependent manner (*n* = 6). (**B**,**C**) 50 μg/mL of clarithromycin pretreatment was administered for 2 h before stimulation with *P. intermedia* supernatants. *CXCL8* mRNA expression post 4 h (**B**) and IL-8 production post 24 h incubation (**C**) were inhibited by clarithromycin pretreatment (*n* = 4−6). Data are presented as mean +/− standard error of the mean (SEM), and each figure is representative from two independent experiments. Significant differences are determined using one-way analysis of variance (ANOVA), followed by Tukey’s multiple comparisons test. *, *p* < 0.05; **, *p* < 0.01; ****, *p* < 0.0001; CAM, clarithromycin.

We performed real-time reverse transcription–polymerase chain reaction (RT-PCR) analysis, which revealed significant enhancement in C-X-C motif chemokine ligand 8 (*CXCL8*) messenger RNA (mRNA) expression after stimulation with *Prevotella* supernatants, indicating a direct concentration-dependent effect (Figure 1A). Furthermore, an expected trend toward suppression of *CXCL8* mRNA expression was observed upon pre-administration of 50 μg/mL of clarithromycin to airway epithelial cells 2 h before stimulation with 50 μg/mL of *Prevotella* supernatant for 4 h (Figure 1B). In addition, IL-8 levels, as measured using enzyme-linked immunosorbent assay (ELISA) post 24 h incubation, mirrored the trends observed at the mRNA level (Figure 1C). These findings strongly suggest that *Prevotella* supernatant acts on human airway epithelium to stimulate neutrophil migration to the lungs and that clarithromycin exerts a definitive inhibitory effect.

### 2.2. Prevotella intermedia Supernatant Stimulates the Expression of CXCL8 mRNA from the Airway Epithelium by Signaling Mitogen-Activated Protein Kinase Kinase 1/2

**Figure 2:**
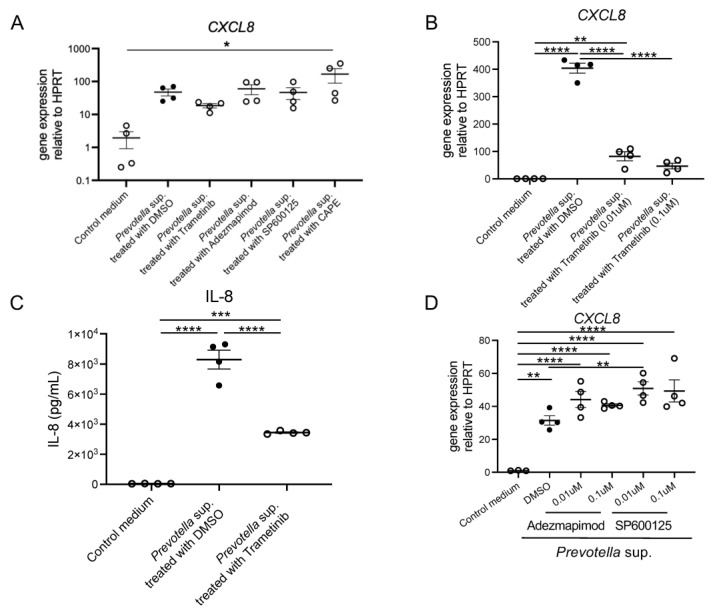
*CXCL8* mRNA expression and IL-8 production by *P. intermedia* supernatants might be induced through transduction via ERK 1/2. (**A**) Inhibition of *CXCL8* mRNA expression with dimethyl sulfoxide, Trametinib, Adezmapimod, SP600125, and caffeic acid phenethyl ester at a concentration of 10 μM was analyzed using real-time RT-PCR post 4 h incubation (*n* = 4). (**B**) *CXCL8* mRNA expression was inhibited by Trametinib pretreatment in a concentration-dependent manner (*n* = 4). (**C**) IL-8 production post 24 h incubation was inhibited by Trametinib pretreatment (*n* = 4). (**D**) *CXCL8* mRNA expression was not influenced by Adezmapimod or SP600125 pretreatment in a concentration-dependent manner (*n* = 4). Data are presented as mean +/− SEM, and each figure is representative from two independent experiments. Significant differences are determined using one-way ANOVA, followed by Tukey’s multiple comparisons test. *, *p* < 0.05; **, *p* < 0.01; ***, *p* < 0.001; ****, *p* < 0.0001; DMSO, dimethyl sulfoxide; Trametinib, MEK 1/2 inhibitor; Adezmapimod, p38 MAPK inhibitor; SP600125, c-JUN N-terminal kinase inhibitor II; CAPE, NF-kβ inhibitor.

Airway epithelial cells were treated with 10 µM of various mitogen-activated protein kinase (MAPK) and nuclear factor kappa B (NF-κβ) inhibitors for 2 h. Subsequently, the cells were stimulated with 50 µg/mL of *Prevotella* supernatant, and *CXCL8* mRNA expression was compared using real-time RT-PCR. The results showed that *CXCL8* mRNA expression was only suppressed in cells pretreated with trametinib (extracellular signal-regulated kinases (ERK) 1/2 inhibitor) (Figure 2A). Notably, *CXCL8* mRNA expression was significantly upregulated in cells pretreated with caffeic acid phenethyl ester (CAPE; NF-κβ inhibitor). The detailed mechanism underlying this finding is unclear; however, it is speculated that the lack of signaling to NF-κB may have facilitated the signaling process. Also, *CXCL8* mRNA expression was inhibited by Trametinib pretreatment in a concentration-dependent manner, suggesting the efficacy is concentration-dependent (Figure 2B). Similarly, we confirmed that Trametinib pretreatment inhibited IL-8 production post 24 h incubation (Figure 2C), whereas Adezmapimod or SP600125 pretreatment did not (Figure 2D).

### 2.3. Prevotella intermedia Supernatant Specifically Induces the Phosphorylation of ERK1/2, Not NF-κB, and the Phosphorylation Is Inhibited by Clarithromycin

**Figure 3:**
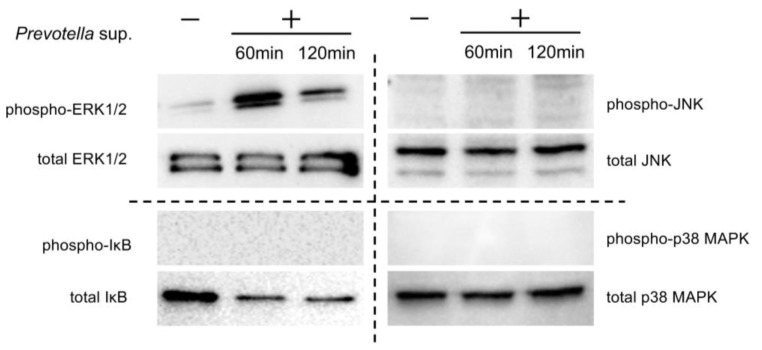
*P. intermedia* supernatants specifically induce the phosphorylation of ERK1/2 rather than other MAPKs and NF-kB. Western blot analyses under reducing conditions with 2.5% 2-mercaptoethanol of each MAPK and NF-kβ in the cell lysates 60 or 120 min post-stimulation with *P. intermedia* supernatants are shown (representative from two independent experiments).

**Figure 4:**
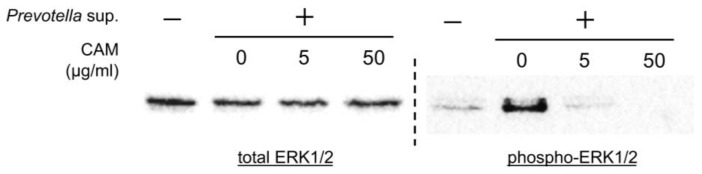
Clarithromycin inhibited ERK1/2 phosphorylation induced by *P. intermedia* supernatants in a concentration-dependent manner. Western blot analyses under reducing conditions with 2.5% 2-mercaptoethanol of total or phosphorylated ERK1/2 in the cell lysates 60 min post-stimulation with *P. intermedia* supernatants under each concentration of clarithromycin treatment are shown (representative from two independent experiments). CAM, clarithromycin.

Western blotting was performed using 30 μg of cell lysates collected 60 and 120 min after stimulation with 50 μg/mL of *Prevotella* supernatant. The phosphorylation of ERK1/2, not p38, c-JUN N-terminal kinase (JNK), or NF-kβ, was observed (Figure 3). Furthermore, Western blotting was performed using cell lysates of airway epithelial cells after 2 h of clarithromycin administration and 60 min of stimulation with 50 μg/mL of *Prevotella* supernatant. We found that ERK1/2 phosphorylation was inhibited by clarithromycin in a concentration-dependent manner (Figure 4). These results suggest that clarithromycin suppresses *CXCL8* mRNA expression by *Prevotella* supernatants by inhibiting ERK1/2 phosphorylation.

### 2.4. CXCL8 mRNA Expression Induced by Prevotella intermedia Supernatant Is Upregulated While ERK3 Is Being Knocked Down

**Figure 5:**
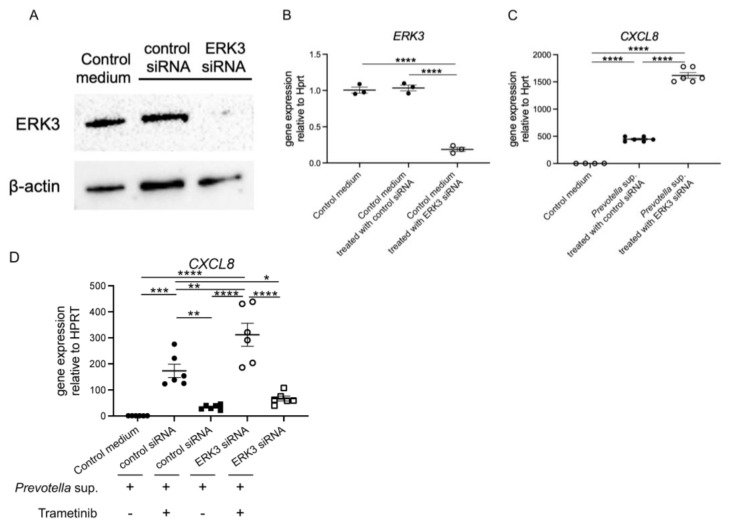
ERK3 knockdown upregulated *CXCL8* mRNA expression induced by *P. intermedia* supernatants. After being transfected with 50 nmol/L of ERK3/MAPK6 small interfering RNAs (siRNAs) or control siRNAs for 72 h, the H292 cells were harvested to confirm the efficiency using (**A**) Western blotting and (**B**) real-time RT-PCR. (**C**) Then, the cells were exposed to *P. intermedia* supernatants for 4 h, and *CXCL8* mRNA expression was assessed using real-time RT-PCR. (**D**) The cells were additionally treated with trametinib, and *CXCL8* mRNA expression was assessed similarly. Data are presented as mean +/− SEM. Significant differences are determined using one-way ANOVA, followed by Tukey’s multiple comparisons test. *, *p* < 0.05; **, *p* < 0.01; ***, *p* < 0.001; ****, *p* < 0.0001.

We reduced the activity of ERK3 in human airway epithelial cells using ERK3/MAPK6 small interfering RNAs (siRNAs) technology. The efficiency was assessed using Western blotting (Figure 5A) and real-time RT-PCR (Figure 5B). Subsequently, we exposed the cells to 50 μg/mL of *Prevotella* supernatant for 4 h and performed real-time RT-PCR analysis of *CXCL8* mRNA expression. Unexpectedly, *CXCL8* mRNA expression was significantly increased in the knockdown condition (Figure 5C), indicating that ERK3 plays a significant role in regulating *CXCL8* mRNA expression. Lastly, we evaluated the effectiveness of trametinib under ERK3 deficiency and confirmed the reduction in *CXCL8* mRNA expression in the ERK3 knockdown condition (Figure 5D). Our findings suggest a negative feedback mechanism in the cells in which *CXCL8* mRNA expression was suppressed, which is contrary to the findings of previous reports.

## 3. Discussion

The characteristics of anaerobes vary across species, even though they are all biophilic anaerobes. The characteristic of *Prevotella* spp., rather than their pathogenicity, might be worsening concurrent pathogen infections, as demonstrated by the mechanism of NET degradation by the bacterial nucleases [6]. *P. intermedia* metabolites have been shown to induce severe neutrophilic inflammation in several preclinical studies [13]. *Prevotella* spp. stimulate epithelial cells to produce IL-8, IL-6, and CCL20, leading to chronic inflammation through the promotion of mucosal Th17 immune responses and neutrophil mobilization, and are associated with various local and systemic diseases [6]. In addition, the ability of *Prevotella* supernatants to induce neutrophil aggregation in the mouse lung has been unequivocally demonstrated in previous preclinical studies [13]. Controlling *Prevotella* spp. may lead to the development of new treatments for various persistent diseases. In the present study, we investigated the mechanism of *CXCL8*/IL-8 over-response and the inhibitory effect of macrolide drugs on pathological exacerbations by stimulating human airway epithelial cells with *Prevotella* culture supernatant and subsequently treating them with macrolide drugs.

In the present study, we found that *P. intermedia* culture supernatant promoted *CXCL8* transcription and IL-8 production in human airway epithelial cells primarily through ERK1/2. This process was effectively suppressed by clarithromycin, a macrolide drug, prohibiting the transduction of ERK1/2. In the previous studies, Mucin-5AC was inhibited by macrolides through the suppression of ERK1/2 transduction [16], and the induction of *CXCL8*/IL-8 expression by *P. intermedia* supernatant might be inhibited by clarithromycin. Recent studies have focused on the maintenance and induction of IL-8 through ERK3 [17]; however, its role in regulating physiological processes, such as innate immunity, remains unclear. In the present study, ERK3 knockdown unexpectedly enhanced *CXCL8* transcription and IL-8 production. This might indicate that ERK1/2 is the primary signaling pathway involved in the production of IL-8 from airway epithelial cells induced by *P. intermedia* supernatant and that signaling to ERK3 might play a crucial role in negative feedback.

Macrolides are one of the most promising candidates for correcting the dysregulation of neutrophilic inflammation induced by the metabolites of *Prevotella* spp. The efficacy of macrolides in treating various diseases, such as bronchiectasis [11], cystic fibrosis [18], and chronic obstructive pulmonary disease, has been demonstrated in recent clinical studies [14,19]. In a recent multicenter prospective study from Australia and New Zealand, it was reported that administering azithromycin thrice weekly to infants diagnosed with cystic fibrosis until the age of 36 months resulted in significant reductions in neutrophilic inflammation, prolonged hospital days due to pulmonary exacerbations, and use of inhalation or oral antibiotics. [18]. Furthermore, it has been reported in other studies that *Prevotella* spp. is involved in the exacerbation of diseases, particularly cystic fibrosis [8,9]. The results of these clinical studies may indirectly focus on the effect of macrolide drugs on *P. intermedia*. In the present study, we focused on the metabolites of *P. intermedia*, as recent advances in Omics analysis have shown that *Prevotella* spp. metabolites are associated with COVID-19 [20] and cystic fibrosis [21] and may be associated with the exacerbation of various unknown diseases. Exacerbation of pneumonia caused by concurrent pathogens due to *Prevotella* spp. has been reported as far back as the 1990s [22]; however, recent advances in genetic diagnosis have gradually shown that the disease burden of mixed infection with *P. intermedia* in cases of community-acquired pneumonia is relatively high [12]. The use of macrolide drugs for severe community-acquired pneumonia has been recommended by the American Thoracic Society/Infectious Diseases Society of America guidelines [23], which is attributed to their various immunomodulatory effects [15]. Based on our findings, the suppression of virulence factors, especially excessive neutrophilic inflammation from *P. intermedia* metabolites by macrolides might be one of the underlying mechanisms, considering the close relationship between *P. intermedia* and pneumonia.

This study has some limitations. First, we used a cell line as human airway epithelial cells, not primary cells, which might have skewed the results. Second, other chemokines in addition to *CXCL8* were not analyzed in this study. Third, we only used *P. intermedia* without other *Prevotella* spp., which could limit the generalizability of the results to other species. Future studies in which various microbes in human primary epithelial cells are utilized are warranted. Fourth, we have not identified causable matters in the supernatants, which remains to be resolved in future research.

## 4. Materials and Methods

### 4.1. Reagents

Clarithromycin was purchased from Merck (PHR1038, Darmstadt, Germany) and diluted in dimethyl sulfoxide (DMSO) purchased from Sigma-Aldrich (D8418, St. Louis, MO, USA) to prepare a stock solution. The following antibodies were supplied by Cell Signaling Technology (Danvers, MA, USA): anti-p44/42 MAPK (Erk1/2) antibody (#9102), anti-Phospho-p44/42 MAPK (Erk1/2) (Thr202/Tyr204) antibody (#9101L), anti-p38 MAPK antibody (#9212), anti-Phospho-p38 MAPK (Thr180/Tyr182) (3D7) rabbit monoclonal antibody (mAb) (#9215), anti-stress-activated protein kinase (SAPK)/JNK antibody (#9252), anti-phospho-SAPK/JNK (Thr183/Tyr185) antibody (#9251), anti-I-kappa-B-alpha (IkBa) (44D4) rabbit mAb (#4812), anti-Phospho-IkBa (Ser32) (14D4) rabbit mAb (#2859), and Erk3 antibody (#4067). Goat anti-rabbit Immunoglobulin G (IgG) (H + L) secondary antibody, horseradish peroxidase (HRP) conjugate (ThermoFisher, A16096, Waltham, MA, USA), and Anti-β actin HRP conjugate (Abcam, ab49900, Waltham, MA, USA) were used as the secondary antibodies. The following inhibitors were diluted in DMSO: Trametinib DMSO solvate (Selleck, S4484, Houston, TX, USA), Adezmapimod (SB203580) (Selleck, S1076), JNK inhibitor II (SP600125) (Selleck, S1460), and CAPE, a specific NF-kβ inhibitor (CAPE; Selleck, S7414).

### 4.2. Preparation of Prevotella intermedia Supernatant

*P. intermedia* used in the study were clinical isolates from a periodontal pocket of a Japanese patient with periodontitis and identified by analyzing whole genome sequences. The *P. intermedia* supernatant was prepared as previously reported [13]. The anaerobe was incubated using modified Gifu Anaerobic Broth (GAM) for 48 h in an anaerobic chamber until the stationary phase. Then, the supernatants were collected through centrifugation at 2000× *g* under 4 °C for 30 min to remove the bacteria and were filtered using a 0.22 mm Millex-GP filter (Millipore, Burlington, MA, USA). We confirmed no bacteria in the supernatant by cultivating the supernatant itself and found no bacteria grown. Protein concentration was analyzed by using Pierce™ BCA Protein Assay Kits (ThermoFisher, Waltham, MA, USA). The supernatant was allocated and stored at −80 °C until use.

### 4.3. Cell Culture

NCI-H292 (ATCC CRL-1848) cells, a human airway epithelial cell line, were cultured in Roswell Park Memorial Institute 1640 medium (ThermoFisher, Waltham, MA, USA) supplemented with 10% fetal bovine serum in a 24-well plate (1 × 10^5^ cells /well). The cells were cultured at 37 °C under 5% carbon dioxide. When the cells reached confluence, they were serum starved for 24 h and then stimulated with 100 μg/mL of *P. intermedia* supernatant. For inhibition, the cells were pretreated with 50 μg/mL of clarithromycin 2 h prior and simultaneously stimulated with *P. intermedia* supernatant. Control media were incubated with a volume of modified GAM equivalent to the volume of *P. intermedia* supernatant owing to the risk of IL-8 production by modified GAM. Cells were also pretreated with signal transduction inhibitors at a concentration of 10 μM for 120 min before stimulation. Cells in control media were incubated with a medium plus the same amount of DMSO without the inhibitors.

### 4.4. Enzyme-Linked Immunosorbent Assay

As described above, NCI-H292 cells were stimulated with 100 μg/mL of *P. intermedia* supernatant, and the culture medium was collected. The secreted protein concentration of IL-8 was measured using enzyme-linked immunosorbent assay (ELISA). The assay was conducted according to the manufacturer’s instructions (IL-8, Human, ELISA Kit, Quantikine, 3rd Generation, Cat# D8000C, R&D Systems, Minneapolis, MN, USA).

### 4.5. Real-Time Quantitative Reverse Transcription-PCR

RNA was isolated using RNeasy Plus Mini Kit (QIAGEN, 74134, Hilden, Germany) thoroughly or post-phase separation using Trizol reagent (ThermoFisher, 15596018), and complementary DNA was prepared using iScript reverse transcriptase master mix (Bio-Rad, 1708841, Hercules, CA, USA). Real-time quantitative RT-PCR was performed using TaqMan PCR master mix (Applied Biosystems, Life Technologies, ThermoFisher, 4369510) and *CXCL8* premixed primers/probe sets (CXCL8 Hs00174103_m1) from Thermo Fisher Scientific (ThermoFisher, 4331182).

### 4.6. Western Blotting

After removing the culture supernatants, cell lysates were dissolved in 50 mg/mL of radioimmunoprecipitation Assay buffer (Cell Signaling Technology, #89900) containing protease inhibitor cocktails (Merck, P8340). The bicinchoninic acid assay was performed to quantify the protein, and 30 μg of the protein was used for Western blotting. Western blotting was performed using 10.0% sodium dodecyl sulfate–polyacrylamide gel electrophoresis gels (Bio-Rad, 4561033) under the reducing condition with 2.5% 2-mercaptoethanol (Bio-Rad, 1610710) and transferred to polyvinylidene difluoride (PVDF) membranes (Bio-Rad, 1704158). After blocking the membrane in 5% skim milk and 0.1% Tween 20 in Tris-buffered saline for 1 h at room temperature, blots were hybridized overnight at 4 °C with primary antibodies. Then, the blots were probed by incubation with goat anti-human IgG-HRP (ThermoFisher, A16096), and the membranes were washed and incubated with SuperSignal West Pico PLUS Chemiluminescent Substrate (Thermo Scientific, 34580). The signal was detected using the Bio-Rad ChemiDoc MP imaging system.

### 4.7. RNA Interference Experiments

RNA interference technology was used to knock down ERK3 expression in NCI-H292 cells. Efficiency and specificity were validated using Western blotting. NCI-H292 cells were cultured in 24-well plates (1 × 10^5^ cells per well) and transfected with 50 nmol/L of ERK3/MAPK6 siRNAs according to the manufacturer’s instructions (Invitrogen, Carlsbad, CA, USA). Transfected cells were incubated for 72 h before being stimulated by *P. intermedia* supernatant. Then, *CXCL8* mRNA expression was compared between cells with and without siRNA treatment. Predesigned RNA oligonucleotides for MAPK6-5 (Hs_MAPK6_5, Cat# SI00606025) and MAPK6-6 (Hs_MAPK6_6, Cat# SI00606032) were obtained from QIAGEN.

### 4.8. Quantification and Statistical Analysis

Statistical analysis was performed using GraphPad Prism software version 10.1.1. Statistical significance was set at *p* < 0.05. Comparisons between two normally distributed groups were performed using a simple two-tailed unpaired Student’s *t*-test. One-way ANOVA with Tukey’s post hoc analysis was used for multiple group comparisons. The normality was determined by the Shapiro–Wilk test. Data were presented as means ± standard error of the mean. *p*-values are annotated as follows: (*) ≤ 0.05, (**) ≤ 0.01, (***) ≤ 0.001, and (****) ≤ 0.0001.

## 5. Conclusions

Our results indicate that clarithromycin may improve the neutrophilic inflammation caused by *P. intermedia* by inhibiting the ERK1/2 transduction and suppressing the exacerbation of concurrent pneumonia. This study provides a reasonable basis for the beneficial effects of macrolides in various clinical settings, particularly cases of lung infections with severe neutrophilic inflammation. Further studies may be necessary to confirm the findings of this study.

## Data Availability

The generated data sets are available from the corresponding author upon reasonable request.
